# Maternal pregnancy hormone profiles in areas with a different incidence of breast cancer

**DOI:** 10.1054/bjoc.2000.1451

**Published:** 2000-11

**Authors:** A A Akhmedkhanov, L Zhu, P G Toniolo

**Affiliations:** Department of Obstetrics and Gynecology, New York University School of Medicine, 550 First Avenue, NB 9E2, New York, NY, 10016, USA


*British Journal of Cancer* (2000) **83**(9), 1255–1259
© 2000 Cancer Research Campaign
doi: 10.1054/ bjoc.2000.1451, available online at http://www.idealibrary.com on IDEAL®

Letters to the Editor

### Maternal pregnancy hormone profiles in areas with a different incidence of breast cancer

**Sir**

In the January 1999 issue of the Journal, Lipworth et al (1999) reported that during the second and third trimester of pregnancy, Chinese women in Shanghai, China had significantly higher levels of several gestational hormones (oestrogens, prolactin, growth hormone) than Caucasian women in Boston, USA (Lipworth et al, 1999). The study indicates that women living in low-risk areas for breast cancer may have hormonal profiles during pregnancy that would stimulate the maturation of mammary cells to a fuller extent, as compared to women in high-risk areas.

A few aspects of the study make difficult its interpretation. Chinese women in the study were on average 6 years younger than American women (mean 25.1 vs 31.0 respectively). Although the literature on the relationships between maternal age, parity and pregnancy hormones is limited, existing evidence suggests that maternal age is an important predictor of pregnancy oestrogen levels. It has been shown that during the third trimester of pregnancy, total oestrogen and oestradiol levels are highest among women aged 20–24, intermediate among women older than 25, and lowest among women below age 20 (Panagiotopoulou et al, 1990). Lipworth et al stated that their results remained essentially unchanged after controlling for maternal age but these data were not reported. It would have been more informative if the results had been presented stratified by age.

Parity is another factor that could affect hormone levels during pregnancy. There is evidence that maternal levels of free oestradiol are higher in the first, as compared to the second, pregnancy (Bernstein et al, 1986). Therefore, reporting the results stratified by parity would have been more helpful, especially considering that 36.8% of American women in the study had a previous full-term pregnancy, as compared to only 3% of Chinese women.

If the authors could provide convincing evidence that the ample differences in maternal age and parity between the two populations have been taken into account appropriately, the study by Lipworth et al raise the interesting possibility that women in an area with a low incidence of breast cancer have a distinct pregnancy hormone profile characterized by higher levels of oestrogens and other mammotrophic hormones. Such hormone profile could advance the development and full maturation of breast cells making them less susceptible to carcinogenesis, as postulated by Russo et al (1982; 1994). In this connection, it would have been of particular interest to have measured pregnancy hormones promoting breast differentiation, such as human chorionic gonadotropin (Russo et al, 1990) and relaxin (Bani et al, 1986).

*AA Akhmedkhanov, L Zhu and PG Toniolo*
*Department of Obstetrics and Gynecology, New York University School of Medicine, 550 First Avenue, NB 9E2, New York, NY 10016, USA*

### REFERENCES

Bani G, Bigazzi M and Bani D (1986) The effects of relaxin on the mouse mammary gland. II. The epithelium. *J Endocrinol Invest* **9**: 145–152

Bernstein L, Depue RH, Ross RK, Judd HL, Pike MC and Henderson BE (1986) Higher maternal levels of free estradiol in first compared to second pregnancy: early gestational differences. *J Natl Cancer Inst* **76**: 1035–1039

Lipworth L, Hsieh CC, Wide L, Ekbom A, Yu SZ, Yu GP, Xu B, Hellerstein S, Carlstrom K, Trichopoulos D and Adami HO (1999) Maternal pregnancy hormone levels in an area with a high incidence (Boston, USA) and in an area with a low incidence (Shanghai, China) of breast cancer. *Br J Cancer* **79**: 7–12

Panagiotopoulou K, Katsouyanni K, Petridou E, Garas Y, Tzonou A and Trichopoulos D (1990) Maternal age, parity, and pregnancy estrogens. *Cancer Causes Control* **1**: 119–124

Russo J, Tay LK and Russo IH (1982) Differentiation of the mammary gland and susceptibility to carcinogenesis. *Breast Cancer Res Treat* **2**: 5–73

Russo IH, Koszalka M and Russo J (1990) Effect of human chorionic gonadotropin on mammary gland differentiation and carcinogenesis. *Carcinogenesis* **11**: 1849–1855

Russo J and Russo IH (1994) Toward a physiological approach to breast cancer prevention. *Cancer Epidemiol Biomarkers Prev* **3**: 353–364

doi: 10.1054/ bjoc.2000.1452, available online at http://www.idealibrary.com on IDEAL®

### Maternal pregnancy hormone profiles in areas with a different incidence of breast cancer – reply

**Sir**

We thank Dr Akhmedkhanov and colleagues for their useful ideas about human chorionic gonodotropin and relaxin and their thoughtful and sensible biologic considerations. In particular, the idea that high levels of hormones during pregnancy might stimulate early breast cell maturation is one of the explanations we are considering for the intrauterine effects on cancer occurrence in the offspring. With respect to the issue of confounding by maternal age and parity, because the findings of higher hormone levels among Chinese women compared with Caucasian women were unexpected for us, we evaluated in detail every possible confounder, including maternal age and parity. When the data were stratified by maternal age (Table 1) or parity (Table 2), Chinese women consistently displayed higher mean levels of the measured hormones at both visits, with the possible exception of progesterone during the second visit, as indicated in our earlier report. (Since confounding does not depend on statistical significance, and in the interest of saving space, we have not presented the confidence interval around the mean.) Thus, we can assure Dr Akhmedkhanov and colleagues, and Journal readers, that there

